# Evaluating Ensemble-Based Machine Learning Models for Diagnosing Pediatric Acute Appendicitis: Insights from a Retrospective Observational Study

**DOI:** 10.3390/jcm14124264

**Published:** 2025-06-16

**Authors:** Zeynep Kucukakcali, Sami Akbulut, Cemil Colak

**Affiliations:** 1 Department of Biostatistics and Medical Informatics, Inonu University Faculty of Medicine, 44280 Malatya, Turkey; zeynep.tunc@inonu.edu.tr (Z.K.);; 2 Department of Surgery and Liver Transplant Institute, Inonu University Faculty of Medicine, 44280 Malatya, Turkey

**Keywords:** pediatric acute appendicitis, machine learning, ensemble learning, diagnostic accuracy, predictive biomarkers

## Abstract

**Background**: Pediatric acute appendicitis (AAP) is a common cause of abdominal pain in children, yet accurate classification into negative, uncomplicated, and complicated forms remains clinically challenging. Misclassification may lead to unnecessary surgeries or delayed treatment. This study aims to evaluate and compare the diagnostic accuracy of five machine learning models (AdaBoost, XGBoost, Stochastic Gradient Boosting, Bagged CART, and Random Forest) for classifying pediatric AAP subtypes. **Methods**: In this retrospective observational study, a dataset of 590 pediatric patients was analyzed. Demographic information and laboratory parameters—including C-reactive protein (CRP), white blood cell (WBC) count, neutrophils, lymphocytes, and appendiceal diameter—were included as features. The cohort consisted of negative (19.8%), uncomplicated (49.2%), and complicated (31.0%) AAP cases. Five ensemble machine learning models (AdaBoost, XGBoost, Stochastic Gradient Boosting, Bagged CART, and Random Forest) were trained on 80% of the dataset and tested on the remaining 20%. Model performance was evaluated using accuracy, sensitivity, specificity, and F1 score, with cross-validation employed to ensure result stability. **Results**: Random Forest demonstrated the highest overall accuracy (90.7%), sensitivity (100.0%), and specificity (61.5%) for distinguishing negative and uncomplicated AAP cases. Meanwhile, XGBoost outperformed other models in identifying complicated AAP cases, achieving an accuracy of 97.3%, sensitivity of 100.0%, and specificity of 78.3%. The most influential biomarkers were neutrophil count, appendiceal diameter, and WBC levels, highlighting their predictive value in AAP classification. **Conclusions**: ML models, particularly Random Forest and XGBoost, exhibit strong potential in aiding pediatric AAP diagnosis. Their ability to accurately classify AAP subtypes suggests that ML-based decision support tools can complement clinical judgment, improving diagnostic precision and patient outcomes. Future research should focus on multi-center validation, integrating imaging data, and enhancing model interpretability for broader clinical adoption.

## 1. Introduction

Acute appendicitis (AAP) is one of the most common causes of acute abdominal pain requiring emergency surgical intervention in both pediatric and adult populations [1,2,3]. As a frequent and significant clinical condition, AAP poses a substantial burden on healthcare systems worldwide. Despite its high prevalence, the accurate diagnosis of AAP remains a considerable challenge due to its variable clinical presentation, which can mimic other abdominal pathologies. This diagnostic complexity is especially evident in atypical cases, where symptoms such as mild abdominal discomfort or non-specific pain may lead to diagnostic uncertainty. Additionally, overlapping symptoms with conditions like gastroenteritis, urinary tract infections, and gynecological disorders further complicate the clinical assessment. Delayed or missed diagnosis of AAP has been associated with an increased risk of severe complications, including perforation, peritonitis, abscess formation, and sepsis. Such complications have been linked to increased morbidity and mortality, as well as longer hospital stays and higher healthcare costs. Conversely, false-positive diagnoses may result in unnecessary appendectomies, which carry potential surgical risks, postoperative complications, and additional healthcare expenditures [4,5]. Pediatric AAP remains one of the most frequently encountered emergencies in pediatric surgery, with an estimated incidence of 86 to 151 cases per 100,000 children annually. It accounts for approximately 1–10% of all pediatric abdominal surgeries and remains a significant cause of morbidity in this population [6,7,8,9,10]. Early and accurate diagnosis is crucial to prevent complications such as perforation and peritonitis.

Traditionally, the diagnosis of AAP relies on a combination of clinical examination, laboratory biomarkers, and imaging techniques. Clinical assessment typically includes evaluating symptoms such as right lower quadrant pain, fever, nausea, and localized tenderness. However, these symptoms alone do not suffice for an accurate diagnosis, as atypical presentations may include vague or diffuse abdominal pain, especially in elderly, pediatric, and pregnant populations. Laboratory markers such as white blood cell count (WBC), C-reactive protein (CRP), and the neutrophil-to-lymphocyte ratio (NLR) are commonly utilized as supportive indicators of inflammation. Elevated WBC and CRP levels, as well as increased NLR, are frequently observed in AAP patients, yet these markers lack specificity as similar elevations can occur in other inflammatory or infectious conditions, such as diverticulitis or pelvic inflammatory disease. Therefore, relying solely on laboratory parameters may lead to diagnostic inaccuracies, especially in ambiguous cases. Imaging techniques are crucial for confirming the diagnosis, particularly when clinical and laboratory findings are inconclusive. Ultrasonography (US) is typically the first-line imaging modality, particularly favored in pediatric and pregnant populations due to its non-invasiveness and absence of ionizing radiation. Despite its advantages, US is highly operator-dependent, and its accuracy may be compromised by factors such as obesity or bowel gas. Computed tomography (CT) remains the most sensitive and specific imaging method for diagnosing AAP in adults, providing detailed visualization of the appendix and surrounding structures. However, concerns regarding radiation exposure limit its routine use in younger patients and pregnant women. Magnetic resonance imaging (MRI) offers a reliable alternative, particularly in pregnant patients, as it avoids radiation. However, its limited availability and prolonged acquisition times often make it less practical in acute emergency settings. Variability in imaging interpretation among practitioners further complicates the diagnostic process, highlighting the need for standardized protocols and training.

Recent advancements in diagnostic strategies have aimed to address these challenges by integrating machine learning (ML) and artificial intelligence (AI) into clinical practice [11]. ML algorithms, known for their ability to process large datasets and recognize complex patterns, have demonstrated significant potential in disease classification and prediction [12,13]. Various studies have explored the application of ML in AAP diagnosis, yielding promising results in enhancing diagnostic precision and minimizing unnecessary surgical interventions. In recent years, explainable ML models incorporating ultrasound imaging have attracted attention for their potential to improve diagnostic processes in cases of suspected AAP. When applied to adult cohorts, these approaches have achieved high diagnostic accuracy in distinguishing AAP from other causes of abdominal pain. On the other hand, two different studies focused on pediatric patients and developed explainable ML approaches that not only predict AAP diagnosis but also management strategies and disease severity. These studies highlight the growing importance of explainable AI in supporting clinical decision-making processes across different age groups [14,15,16].

Despite these promising developments, there remains a gap in the literature regarding the comparative performance of different ML models in pediatric AAP diagnosis [16,17,18,19]. Although individual models like XGBoost and Random Forest (RF) have demonstrated efficacy, there is a lack of comprehensive studies comparing multiple models within the same clinical context. Addressing this gap is crucial, as identifying the most accurate and clinically applicable model could significantly enhance diagnostic workflows and patient outcomes. The objective of this study is to evaluate and compare the diagnostic performance of five ensemble ML algorithms—AdaBoost, XGBoost, Stochastic Gradient Boosting (SGB), Bagged CART, and RF—in distinguishing between negative, uncomplicated, and complicated cases of pediatric AAP. Using routinely collected clinical and laboratory biomarkers, each model was trained and tested to assess its classification accuracy, sensitivity, specificity, and overall clinical utility. By systematically applying and validating these ML models, the study aims to identify the most effective computational approach to support early and accurate diagnosis in pediatric AAP, which may assist in clinical decision-making and potentially reduce the risk of unnecessary surgical interventions.

## 2. Materials and Methods

### 2.1. Dataset

This retrospective observational study utilized an open-access dataset originally collected at the Department of Pediatric Surgery, Charité-Universitätsmedizin Berlin. We included patients younger than 18 years who underwent appendectomy between December 2006 and September 2016 [1]. The dataset comprises information on 590 pediatric patients who underwent surgery for suspected AAP. Medical records were reviewed as part of the dataset creation process, including demographic variables (age and gender) and standard diagnostic parameters such as CRP levels, complete blood cell counts, ultrasound findings (including appendiceal diameter), and postoperative histopathological results.

Inclusion criteria involved pediatric patients with complete clinical and laboratory records and histopathologically confirmed diagnoses categorized as negative, uncomplicated, or complicated AAP. Exclusion criteria included missing histopathology or laboratory data, the presence of chronic comorbidities, secondary or elective appendectomies, and other appendix-related pathologies such as oxyuriasis and carcinoid tumors.

The dataset was curated through retrospective chart review and anonymized prior to public release. As the primary aim of the original study was the development of diagnostic classification models, no postoperative or long-term follow-up data were collected or analyzed. Ethical approval was obtained in the original study. The present work adheres to the STROBE (Strengthening the Reporting of Observational Studies in Epidemiology) guidelines, with explicit reporting of study design, inclusion and exclusion criteria, data collection period, and data sources.

### 2.2. Machine Learning and the Models Used in the Study

ML has developed as a revolutionary technology in multiple fields, allowing computers to learn from data and enhance their performance progressively [20]. The practical applications of ML are extensive and diverse. Healthcare professionals utilize ML algorithms to identify diseases, predict patient outcomes, and tailor treatment approaches. Research has shown the effectiveness of ML in identifying illnesses, including melanoma and heart disease, highlighting its capacity to improve clinical decision-making [21,22,23]. The capacity of ML to swiftly and precisely assess extensive datasets renders it an indispensable asset in these scenarios. There are many ML algorithms. This study employed AdaBoost, XGBoost, Stochastic Gradient Boosting, Bagged CART, and RF algorithms. All of these algorithms are leading ensemble learning techniques with unique features and applications in ML.

In this study, the primary outcome was the diagnostic performance of five ensemble-based ML algorithms—AdaBoost, XGBoost, Stochastic Gradient Boosting (SGB), Bagged Classification and Regression Trees (CART), and RF—in classifying pediatric AAP cases into negative, uncomplicated, and complicated categories. Model performance was assessed separately for each binary classification task using standard evaluation metrics, including accuracy, sensitivity, specificity, positive predictive value (PPV), negative predictive value (NPV), and F1 score.

As secondary outcomes, the most relevant clinical and laboratory features contributing to model predictions were identified through variable importance analysis. Feature importance values were computed within each algorithm and normalized by assigning a score of 100 to the most influential variable, enabling relative comparisons among all predictors. These normalized scores provided insights into which biomarkers—such as neutrophil count, appendiceal diameter, and CRP—had the greatest impact on the classification of AAP subtypes.

### 2.3. AdaBoost (Adaptive Boosting)

AdaBoost (Adaptive Boosting) is an ensemble technique that integrates several weak classifiers to form a robust classifier. AdaBoost operates by sequentially implementing weak classifiers on the training data, focusing on cases misclassified in previous iterations. This iterative procedure modifies the weights of the training samples, assigning greater significance to those that are challenging to categorize. AdaBoost has demonstrated considerable efficacy across several applications, attaining high accuracy in tasks including face detection and the classification of imbalanced datasets [24,25].

### 2.4. XGBoost (Extreme Gradient Boosting)

XGBoost (Extreme Gradient Boosting) is a sophisticated implementation of gradient boosting that enhances both speed and performance. It utilizes a gradient descent approach to reduce the loss function, rendering it very efficient for extensive datasets. XGBoost employs regularization approaches to mitigate overfitting; hence, it improves its generalization abilities relative to conventional boosting algorithms [26].

### 2.5. SGB (Stochastic Gradient Boosting)

SGB is a form of gradient boosting that incorporates randomness into the model training procedure. Randomly selecting a data subset for each iteration mitigates the danger of overfitting and enhances the model’s robustness. This stochastic method facilitates expedited training durations and may enhance generalization on unfamiliar material [26].

### 2.6. Bagged CART (Bagged Classification and Regression Trees)

Bagged CART (Bagged Classification and Regression Trees) is an ensemble technique that uses bootstrap aggregating (bagging) to enhance the stability and precision of decision trees. This method involves training several decision trees on various data subsets, with their predictions averaged for regression or subjected to voting for classification to generate a final output. Bagging diminishes variance and alleviates overfitting, rendering it a reliable option for numerous classification and regression problems [26].

### 2.7. Random Forest (RF)

RF is an enhancement of the bagging method that constructs numerous decision trees and consolidates their results. In contrast to Bagged CART, RF incorporates extra randomization by choosing a random subset of characteristics for each split in the decision trees. This feature selection technique increases variety within the trees, resulting in enhanced accuracy and resilience against overfitting [26]. In conclusion, the ensemble learning models utilized in this study—AdaBoost, XGBoost, Stochastic Gradient Boosting (SGB), Bagged CART, and RF—are designed to improve predictive accuracy and model stability by combining multiple base learners.

AdaBoost works by sequentially training weak classifiers, typically decision stumps, and adjusting the weights of misclassified instances in each iteration to focus on the most challenging cases. XGBoost and Stochastic Gradient Boosting (SGB) both build trees sequentially, with each new tree trained to correct the residual errors of the previous ensemble. They use gradient descent optimization to minimize a loss function, allowing them to model complex, non-linear relationships. XGBoost adds regularization to prevent overfitting and is highly efficient in handling sparse data. Bagged CART generates multiple versions of a decision tree using bootstrap sampling and aggregates their predictions (typically by majority voting for classification) to reduce variance and enhance generalization. RF extends bagging by adding random feature selection at each split in the decision tree, which increases model diversity and further reduces overfitting.

Overall, these models function by combining the strengths of individual learners—either sequentially (boosting) or in parallel (bagging)—to build more accurate and generalizable classifiers. Their collective ability to handle high-dimensional data, capture complex feature interactions, and reduce variance or bias makes them particularly suitable for clinical classification tasks such as differentiating AAP subtypes.

### 2.8. Modeling Phase

We divided the data set into 80% training data and 20% test data for the mentioned models. This study employed the n-fold cross-validation technique, a resampling method, to ascertain the model’s validity. The n-fold cross-validation technique involves dividing the dataset into *n* subsets and then applying the model to each subset. In the next step, we allocate one component from the total of *n* components for testing and use the remaining *n* − 1 components for training. We assess the cross-validation approach in the last stage by computing the mean of the values obtained from the models. We assessed the modeling performance using various metrics such as accuracy (ACC), balanced accuracy (b-ACC), sensitivity (SE), specificity (SP), PPV, NPV, and F1 score. Finally, the modeling yielded variable importance values, allowing us to identify the variables that have the greatest influence on the target. Variable importance scores were normalized such that the most influential feature in each model was scaled to 100, and all other features were assigned values relative to this maximum.

### 2.9. Study Protocol and Ethics Committee Approval

This observational study involving human participants utilized an open-access dataset and was conducted in full compliance with both institutional and national ethical standards, including the principles of the 1964 Declaration of Helsinki and its subsequent revisions or equivalent ethical guidelines. Ethical approval was obtained from the Inonu University Institutional Review Board (IRB) for non-interventional clinical research (Approval No: 2025/7441). The methodological rigor and risk of bias were assessed using the STROBE (Strengthening the Reporting of Observational Studies in Epidemiology) statement [27].

### 2.10. Biostatistical Analyses

We used IBM SPSS Statistics for Windows, version 25.0 (IBM Corporation, Armonk, NY, USA), for statistical analysis. We summarized the data in the study using the median (95% confidence interval) and count (percentage). Normality was checked with Kolmogorov Smirnov. We used the Mann–Whitney U test to compare the groups. In statistical analyses, *p* < 0.05 was considered significant.

## 3. Results

The study analyzed data from 590 pediatric patients who underwent surgery with a diagnosis of AAP. Table 1 presents the distribution of the patients’ gender and their classification into respective groups. Table 1 shows that 80.2% of the patients received an AAP diagnosis, while 19.8% received a negative diagnosis. Among the AAP cases, subgroup analysis revealed that 31.0% of patients had a complicated AAP, whereas 49.2% had an uncomplicated AAP. As previously noted, the negative AAP rate was 19.8%. Gender distribution analysis indicated that males comprised 54.9% of the cohort, while females accounted for 45.1%.

Table 2 provides a detailed overview of the demographic characteristics and biochemical blood parameters of the patients. The median age of the patients was 11 years, with a confidence interval level (95% CI) ranging from 11 to 12 years. Evaluation of laboratory findings yielded the following median values: CRP at 12.5 mg/L (95% CI: 9.7–15.5), platelets at 272 × 10^9^/L (95% CI: 265–279), WBC at 13.4 × 10^9^/L (95% CI: 13.1–14.1), neutrophils at 10.7 × 10^9^/L (95% CI: 10.4–11.2), immature granulocytes at 0.04 × 10^9^/L (95% CI: 0.04–0.05), lymphocytes at 1.62 × 10^9^/L (95% CI: 1.52–1.68), monocytes at 0.92 × 10^9^/L (95% CI: 0.88–0.96), eosinophils at 0.04 × 10^9^/L (95% CI: 0.04–0.06), and basophils at 0.03 × 10^9^/L (95% CI: 0.03–0.04). Additionally, we measured the median diameter of the appendix vermiformis at 9 mm (95% CI: 9–10 mm).

Table 3 presents the demographic, clinical, and laboratory data for the negative AAP group and the uncomplicated AAP group. In comparisons between negative and uncomplicated AAP groups, the uncomplicated group exhibited significantly higher values for age, CRP, WBC, neutrophils, immature granulocytes, monocytes, and appendix diameter. In contrast, lymphocytes and eosinophils were significantly elevated in the negative group. No statistically significant differences were found in platelet and basophil counts.

Table 4 indicates the modeling results for negative and uncomplicated AAP groups with the five ML algorithms mentioned. For distinguishing negative vs. uncomplicated AAP, RF achieved the highest overall performance, with 90.7% accuracy and perfect sensitivity.

**Figure 1:**
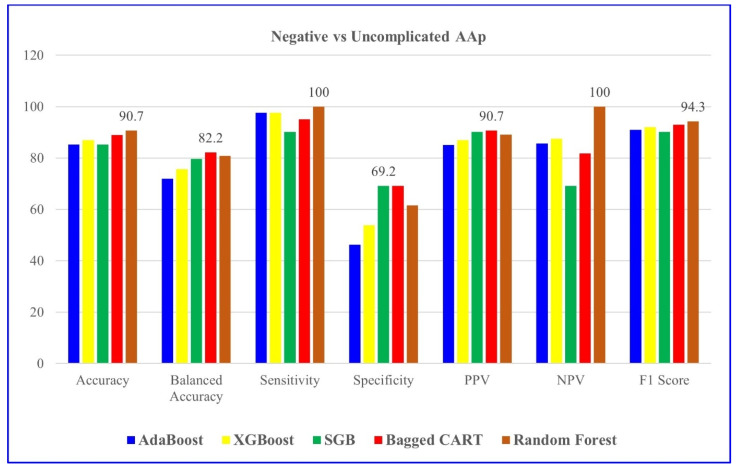
Comparison of negative and uncomplicated AAP groups based on five ML models. SGB: Stochastic Gradient Boosting.

Table 5 evaluates the results of variables’ importance levels in distinguishing negative and uncomplicated AAP groups for RF, the algorithm with the highest model performance. Figure 1 also presents it graphically. Table 5 clearly identifies neutrophils as the most significant factor, with a variable importance value of 100. Appendix diameter, with a value of 94.668, closely follows the neutrophilia, while WBC, with a value of 92.1, is also considered an important variable. The values of CRP, at 69.654, and those of lymphocytes and platelets, at 69.593, indicate a moderate level of importance. Monocytes contribute at a similar level with values of 69.184, and immature granulocytes with values of 67.364. The variables with lower importance values are eosinophils (59.088), age (53.51), platelets (51.716), and basophils (26.395). As a result, neutrophils, appendix diameter, and WBC stand out as the most important determinants in distinguishing negative and uncomplicated AAP. In particular, inflammatory markers and appendix diameter play a critical role in terms of differential diagnosis.

Table 6 presents the demographic, clinical, and laboratory data for the negative AAP group and the complicated AAP group. In comparisons between negative and complicated AAP groups, the complicated group exhibited significantly higher values for age, CRP, platelets, WBC, neutrophils, immature granulocytes, monocytes, and appendix diameter. Conversely, lymphocytes, eosinophils, and basophils were elevated in the negative group.

**Figure 2:**
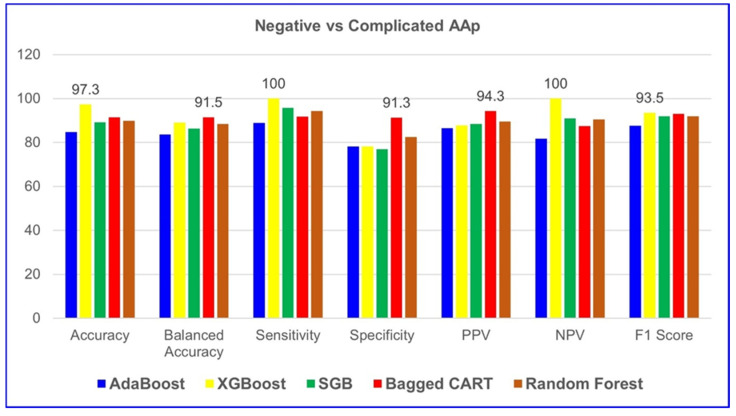
Comparison of negative and complicated AAP groups based on five ML models. SGB: Stochastic Gradient Boosting.

According to Table 7, XGBoost demonstrated the highest performance among all five ML algorithms in classifying negative and complicated AAP groups. The model achieved 97.3% accuracy, 100% sensitivity, 100% NPV, and a 93.5% F1 score. These results indicate that XGBoost provides excellent predictive capability for both positive and negative cases, making it the most reliable model for distinguishing between negative and complicated AAP cases. Table 8 evaluates the results, showing the importance levels of the variables in distinguishing negative and complicated AAP groups for XGBoost, the algorithm with the highest model performance. Figure 2 graphically presents it.

Table 8 reveals that appendix diameter has the highest significance at 100%, indicating its crucial role in differentiating between negative and complicated AAP groups. CRP, at 71.5%, is a significant variable, suggesting that elevated levels of inflammation are indicative of complicated AAP. WBC (56.0%) and neutrophils (44.4%) are also significant parameters that effectively determine inflammation and complications. Other biochemical parameters had lower significance and were less decisive compared to appendix diameter and CRP.

Table 9 presents the demographic, clinical, and laboratory data for the uncomplicated AAP and complicated AAP groups. In comparisons between the two groups, the complicated AAP group exhibited significantly higher values for CRP, WBC, neutrophils, immature granulocytes, monocytes, and appendiceal diameter, as well as age. In contrast, the uncomplicated AAP group showed significantly higher levels of lymphocytes, eosinophils, and basophils. These findings suggest that inflammatory markers and immune cell profiles may play a critical role in differentiating between uncomplicated and complicated AAP cases.

Table 10 shows that XGBoost achieved the best performance in distinguishing uncomplicated and complicated AAP cases, with 80% accuracy, 78.1% balanced accuracy, and a 72.3% F1 score. The model also demonstrated strong predictive ability with 70.8% sensitivity, 85.4% specificity, 73.9% PPV, and 83.3% NPV, making it the most effective algorithm for this classification task.

**Figure 3:**
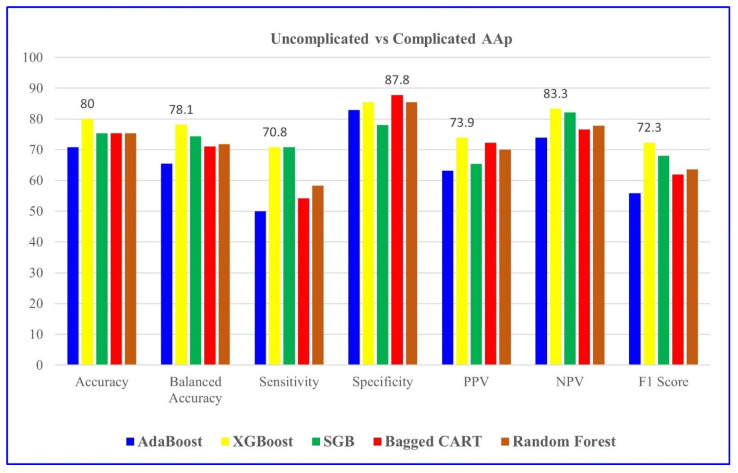
Comparison of uncomplicated and complicated AAP groups based on five ML models. SGB: Stochastic Gradient Boosting.

Table 11 evaluates the results, showing the importance of variables in distinguishing complicated and uncomplicated AAP groups for XGBoost, the algorithm with the highest model performance. Figure 3 graphically presents it. According to the table, CRP stands out as the variable with the highest significance at 100%, indicating that inflammation levels play a crucial role in distinguishing between complicated and uncomplicated AAP groups. Eosinophils (42.8%) and monocytes (19.1%) are among the lower but significant variables, while age (14.0%) and WBC (13.8%) also contribute to determining some differences. These results indicate that CRP is the most critical parameter, with other biochemical variables playing an important role, albeit proportionally much less.

## 4. Discussion

Given the diagnostic complexity and the clinical burden of pediatric AAP, along with the risk of serious complications arising from delayed or inaccurate diagnosis, there is an urgent need for improved and timely diagnostic strategies—particularly in pediatric populations. Early and accurate diagnosis is crucial to prevent complications such as perforation and peritonitis. This underscores the necessity of advancing diagnostic methodologies, particularly through the integration of ML models into clinical workflows to enhance decision-making and efficiency. The evolving role of AI and ML in healthcare has opened new frontiers in diagnostic precision. This study assessed five ML models—AdaBoost, XGBoost, SGB, Bagged CART, and RF—within the context of pediatric AAP. Notably, the RF model exhibited superior performance in distinguishing negative and uncomplicated AAP cases, achieving an accuracy of 90.7%, sensitivity of 100.0%, and specificity of 61.5%. Meanwhile, XGBoost emerged as the most reliable model for classifying complicated AAP cases, with an accuracy of 97.3%, sensitivity of 100.0%, and specificity of 78.3%. These findings underscore the advantages of ensemble learning techniques, which combine multiple weak classifiers to enhance overall predictive accuracy and refine patient stratification. Importantly, key hematological and clinical biomarkers, such as neutrophil count, appendiceal diameter, and WBC count, played a crucial role in predictive accuracy.

Our findings align with and extend the prior literature on ML-driven diagnostic tools in pediatric AAP. Marcinkevičs et al. [16] emphasized the potential of ML models in improving diagnostic accuracy using ultrasound images, showing that deep learning-based segmentation could enhance radiological assessment. Meanwhile, Reismann et al. [1] highlighted the viability of AI-based classification approaches in pediatric AAP diagnosis, but their study was limited to a narrower dataset and lacked a comparative evaluation of different ML models. Unlike these studies, our research provides a direct comparative analysis of multiple ML algorithms applied to a large dataset, allowing a more comprehensive assessment of model performance in real-world clinical scenarios. Most existing studies emphasize single-model implementation, limiting their ability to capture the comparative strengths and weaknesses of various ML approaches. In contrast, our study directly evaluates multiple ML algorithms, offering a broader perspective on their diagnostic capabilities. A comparative approach is essential for identifying the strengths and weaknesses of different models in real-world clinical applications, as no single algorithm performs optimally across all diagnostic tasks. By evaluating multiple models, we provide insights into which algorithms are most suitable for specific patient subgroups and clinical scenarios, ultimately advancing the precision and reliability of ML-based decision support tools in pediatric AAP diagnosis. This approach strengthens the growing evidence that ML-based decision support tools could refine clinical workflows, minimize misdiagnoses, and reduce unnecessary surgical interventions. Nevertheless, while ML models can augment clinical decision-making, it is crucial to recognize that their efficacy is contingent upon data quality, patient demographics, and model optimization.

Recent systematic reviews have consistently demonstrated the effectiveness of ML in the diagnosis of AAP, particularly through the use of laboratory markers such as CRP, WBC, and neutrophil count [28,29]. While Lam et al. [29] systematically reviewed various AI models, including logistic regression, random forests, and neural networks, their study mainly summarized existing models without providing a direct head-to-head comparison of multiple ensemble algorithms. In contrast, our study offers a comparative evaluation of several ensemble-based machine learning algorithms, demonstrating their superior performance in classifying AAP subtypes. This positions our research beyond previous work by not only confirming the importance of inflammatory biomarkers but also validating their predictive value across various advanced ML techniques. Meanwhile, Bianchi et al. [30] reviewed the application of AI techniques in the diagnosis and management of AAP, including studies that integrated machine learning with imaging modalities such as ultrasound and CT, highlighting their potential to improve diagnostic accuracy. Hsieh et al. [31] compared traditional scoring systems such as the Alvarado Score with multiple machine learning-based models, including random forest, support vector machines, artificial neural networks, and logistic regression, demonstrating that ML algorithms, particularly ensemble methods like random forest, achieved superior diagnostic performance (AUC = 0.98 for RF vs. 0.77 for Alvarado). Their findings highlight the potential value of advanced machine learning models over conventional scoring systems in improving the accuracy of AAP diagnosis. However, their study focused primarily on single learner models, random forest and did not incorporate ensemble learning techniques, which have been shown to provide higher accuracy and robustnes. Our findings extend this understanding by demonstrating that ensemble methods such as RF and XGBoost outperform traditional approaches, further solidifying the role of ML in pediatric AAP diagnosis. Chekmeyan et al. [32] discussed challenges in the clinical integration of ML models, stressing the need for improved interpretability and physician acceptance—issues that align with our own observations about the real-world applicability of ML in pediatric AAP. The present study can be compared with the recent work by Males et al. [19], who developed an explainable ML model to reduce the rate of negative appendectomies in pediatric patients with a high pre-test probability of AAP. Their model, based on XGBoost and SHAP, focused on binary classification and decision support in patients already classified as likely to have AAP, aiming to reduce unnecessary surgeries. In contrast, our study addressed the broader challenge of differentiating between negative, uncomplicated, and complicated AAP cases at the time of presentation, using five ensemble learning algorithms and clinical/laboratory variables without incorporating imaging data. While Male et al. [19] prioritized interpretability and clinical trust in high-risk decision-making, our work focused on performance benchmarking across ensemble models and identifying key predictive biomarkers through feature importance analysis. Notably, both studies emphasize the utility of ML in pediatric AAP management and support its integration into clinical workflows. However, our multiclass approach offers additional value in stratifying disease severity, which may inform not only the decision to operate but also the urgency and modality of treatment.

Bianchi et al. [30] emphasized that integrating imaging data with ML models enhances the reliability of diagnostic predictions, favoring hybrid models that combine clinical and radiological features. In contrast to Bianchi et al. [30], our research relies exclusively on readily accessible, cost-effective clinical and hematological biomarkers—such as CRP, WBC, and neutrophil count—without incorporating any imaging data. Despite this, our models achieved comparable or superior diagnostic performance, particularly with ensemble methods like RF and XGBoost. This distinction underscores the novelty and practical significance of our work. It demonstrates that high diagnostic accuracy in pediatric AAP classification can be achieved without the reliance on imaging, which is often operator-dependent, expensive, or unavailable in low-resource settings. By showing that biochemical markers alone are sufficient for effective ML-based diagnosis, our study not only supports but extends the existing literature by providing a cost-efficient and scalable diagnostic alternative. This contribution is especially relevant in emergency and rural clinical environments, where rapid and reliable diagnosis without advanced imaging can significantly impact patient outcomes and healthcare resource allocation. Hsieh et al. [31] demonstrated that machine learning models achieved superior diagnostic performance compared to traditional clinical scoring systems, with random forest emerging as the most accurate and reliable model in distinguishing patients with and without AAP. Finally, Chekmeyan et al. [32] suggested that while ML models show promise, real-world application remains limited by the need for model transparency and clinician trust in AI-driven decision-making.

This study offers several distinctive contributions. First, by comparing five ML models, we provide a more granular understanding of algorithmic efficacy in pediatric AAP diagnosis. Second, the study benefits from a substantial dataset comprising 590 pediatric patients, enhancing generalizability. Third, our integration of hematological and biochemical parameters—such as CRP, WBC, and neutrophil count—demonstrates the potential of ML models in leveraging routinely available laboratory markers for enhanced diagnostic accuracy. Finally, the implementation of cross-validation techniques ensures the reliability and robustness of our findings, distinguishing this study from prior work that lacks rigorous validation frameworks.

### 4.1. Challenges and Limitations

Despite its strengths, this study has inherent limitations that impact the interpretation and applicability of its findings. The single-center nature of the dataset may restrict the generalizability of the results, limiting their applicability to broader populations with varying demographic or clinical characteristics. Additionally, the lack of imaging-based parameters, such as ultrasound or CT findings, constrains the model’s ability to fully mimic real-world clinical decision-making, where such tools are integral.

Although the sample size may appear relatively limited, this study utilized a fully curated and anonymized open-access dataset made publicly available by the Department of Pediatric Surgery, Charité-Universitätsmedizin Berlin. The dataset comprises pediatric patients who underwent appendectomy between December 2006 and September 2016 and represents the finalized and complete version released by the data custodians. No additional patient data beyond 2016—particularly for the years 2017 to 2024—were included in the open-access release and, therefore, were not accessible for analysis. As a result, the study period and sample size were inherently limited by the structure and temporal scope of the publicly accessible dataset, which included only cases recorded between 2006 and 2016. Despite this limitation, the dataset remains one of the largest and most detailed open-access resources for pediatric AAP, allowing for rigorous ML analysis supported by cross-validation to reduce overfitting and improve generalizability.

The absence of comprehensive hyperparameter tuning could also mean that the models have not achieved their optimal performance, potentially affecting their predictive accuracy in different clinical settings. Moreover, the retrospective design and exclusion of incomplete records introduce a degree of selection bias, which may limit the representativeness of the study population.

It is also important to acknowledge the potential for class imbalance, particularly in the distribution of negative, uncomplicated, and complicated AAP cases, which may affect model calibration and contribute to biased predictions toward majority classes.

Furthermore, while ML models demonstrate promising diagnostic performance, their seamless integration into clinical workflows remains a significant challenge. Factors such as the limited interpretability of complex algorithms, variability in physician trust toward automated systems, and the absence of standardized regulatory frameworks continue to hinder their real-world adoption. These issues are further compounded by potential concerns regarding accountability in decision-making, medicolegal implications, and the risk of automation bias in critical care settings. Without transparent and explainable models that can align with clinicians’ diagnostic reasoning, the acceptance and reliability of such tools remain uncertain.

Moreover, the implementation of ML-based tools in diverse clinical environments requires extensive external validation and prospective evaluation. To move from theoretical accuracy to practical utility, future studies should prioritize multi-center data acquisition, incorporate radiological and imaging-based features, and establish protocols for clinical interpretability and feedback. Addressing these barriers will be crucial not only for enhancing model robustness and fairness but also for building the institutional and regulatory trust necessary for successful clinical deployment.

### 4.2. Future Directions: Bridging the Gap Between AI and Clinical Implementation

Future studies should prioritize multi-center validation to improve the external generalizability of these models. Such an approach would enable testing across diverse patient populations and clinical settings, ensuring robustness and minimizing bias. For instance, the FDA’s framework for AI-driven medical tools requires ongoing validation and real-world performance monitoring, which can slow down clinical adoption. Similarly, the European Medicines Agency (EMA) has strict guidelines on AI transparency and bias mitigation, making compliance a significant hurdle for developers. Multi-center validation would help address the limitations associated with single-institution studies by incorporating a broader range of patient demographics, clinical presentations, and healthcare settings. This approach would enhance the generalizability of the models and ensure their robustness across different populations, ultimately facilitating their integration into real-world clinical practice. Incorporating imaging data, deep learning architectures, and more sophisticated feature engineering strategies may further enhance diagnostic accuracy. Additionally, optimizing ML models through hyperparameter tuning and ensemble learning could improve robustness. One key challenge in clinical adoption is the ‘black-box’ nature of ML models, which can make clinicians hesitant to trust AI-driven diagnoses. Explainable AI (XAI) techniques, such as SHAP (Shapley Additive Explanations) or LIME (Local Interpretable Model-Agnostic Explanations), have been proposed to enhance transparency, yet their integration into clinical workflows remains limited. Finally, developing real-time clinical decision support systems that integrate ML models into emergency settings remains an important next step toward practical implementation.

## 5. Conclusions

ML has emerged as a powerful adjunct in the diagnosis of pediatric AAP. However, the successful clinical adoption of ML models faces several barriers. Regulatory challenges, including approval processes and standardization across different healthcare systems, must be addressed to ensure safe and consistent implementation. To facilitate clinical adoption, structured training programs that enhance AI literacy and model interpretation are essential. These initiatives should equip clinicians with the knowledge needed to confidently integrate ML-driven decision support tools into their practice. Without sufficient training, even highly accurate ML models may fail to gain widespread clinical adoption. Additionally, clinician training is a crucial factor, as healthcare providers need to understand and trust ML-driven decision support tools to incorporate them into routine practice. Further research should explore strategies to improve model interpretability and establish guidelines for integrating ML models seamlessly into clinical workflows. Our findings reinforce the potential of ML models—particularly RF and XGBoost—in improving diagnostic precision and minimizing unnecessary surgical interventions. However, successful clinical translation requires continued research, external validation, and thoughtful integration into clinical practice. By addressing these challenges, ML-based diagnostic tools may ultimately enhance patient care, streamline decision-making, and support more efficient resource allocation in emergency settings.

## Figures and Tables

**Table 1:** Qualitative variables of entire study population.

Variables (Median (95% CI))	Categories	Results [*n* (%)]
Main groups	AAP	473 (80.2)
	Negative AAP	117 (19.8)
Subgroups	Complicated AAP	183 (31.0)
	Uncomplicated AAP	290 (49.2)
	Negative AAP	117 (19.8)
Gender	Male	324 (54.9)
	Female	266 (45.1)

AAP: Acute appendicitis, CI: Confidence interval.

**Table 2:** Quantitative variables of entire study population.

Variables (Median (95% CI))	Results
Age (years)	11 (11–12)
CRP (mg/L)	12.5 (9.7–15.5)
Platelets (×10^9^/L)	272 (265–279)
WBC (×10^9^/L)	13.4 (13.1–14.1)
Neutrophils (×10^9^/L)	10.7 (10.4–11.2)
Immature granulocytes (×10^9^/L)	0.04 (0.04–0.05)
Lymphocytes (×10^9^/L)	1.62 (1.52–1.68)
Monocytes (×10^9^/L)	0.92 (0.88–0.96)
Eosinophils (×10^9^/L)	0.04 (0.04–0.06)
Basophils (×10^9^/L)	0.03 (0.03–0.04)
Appendix diameter (mm)	9 (9–10)

CRP: C-reactive protein, WBC: White blood cell count, CI: Confidence interval.

**Table 3:** Negative versus uncomplicated AAP.

Variables (Median (95% CI))	Negative App	Uncomplicated	*p*
Age	13 (13–15)	11 (11–12)	<0.001
CRP	3 (2.5–5.5)	7.9 (6.3–9.7)	<0.001
Platelets	263 (245–281)	274 (264–283)	0.156
WBC	9.1 (8.6–10.5)	13.5 (13.2–14.3)	<0.001
Neutrophils	5.8 (5.1–6.6)	10.7 (10.3–11.5)	<0.001
Immature granulocytes	0.02 (0.02–0.03)	0.05 (0.05–0.06)	<0.001
Lymphocytes	2.07 (1.94–2.29)	1.66 (1.55–1.77)	<0.001
Monocytes	0.73 (0.65–0.79)	0.90 (0.84–0.97)	<0.001
Eosinophils	0.10 (0.08–0.16)	0.05 (0.04–0.07)	<0.001
Basophils	0.03 (0.03–0.04)	0.03 (0.03–0.04)	0.717
Appendix diameter (mm)	7 (7–8)	9 (9–10)	<0.001

CRP: C-reactive protein, WBC: White blood cell count, CI: Confidence interval.

**Table 4:** Modeling results for negative versus uncomplicated AAP.

	AdaBoost	XGBoost	SGB	Bagged CART	Random Forest
Accuracy	85.2	87.0	85.2	88.9	90.7
Balanced Accuracy	71.9	75.7	79.7	82.2	80.8
Sensitivity	97.6	97.6	90.2	95.1	100.0
Specificity	46.2	53.8	69.2	69.2	61.5
PPV	85.1	87.0	90.2	90.7	89.1
NPV	85.7	87.5	69.2	81.8	100.0
F1 Score	90.9	92.0	90.2	92.9	94.3

SGB: Stochastic Gradient Boosting, PPV: positive predictive value; NPV: negative predictive value.

**Table 5:** Variables’ importance for negative versus uncomplicated AAP.

Variables	Variable Importance
Neutrophils	100
Appendix diameter (mm)	94.668
WBC	92.1
CRP	69.654
Lymphocytes	69.593
Monocytes	69.184
Immature granulocytes	67.364
Eosinophils	59.088
Age	53.51
Platelets	51.716
Basophils	26.395

CRP: C-reactive protein, WBC: White blood cell count.

**Table 6:** Negative versus complicated AAP.

Variables (Median (95% CI))	Negative App	Complicated	*p*
Age	13 (13–15)	10 (10–11)	<0.001
CRP	3 (2.5–5.5)	42.6 (35.2–58)	<0.001
Platelets	263 (245–281)	278 (265–295)	0.038
WBC	9.1 (8.6–10.5)	15.3 (14.7–16.5)	<0.001
Neutrophils	5.8 (5.1–6.6)	12.8 (12.0–13.7)	<0.001
Immature granulocytes	0.02 (0.02–0.03)	0.05 (0.05–0.06)	<0.001
Lymphocytes	2.07 (1.94–2.29)	1.25 (1.14–1.4)	<0.001
Monocytes	0.73 (0.65–0.79)	1.1 (1.02–1.18)	<0.001
Eosinophils	0.10 (0.08–0.16)	0.010 (0.01–0.02)	<0.001
Basophils	0.03 (0.03–0.04)	0.02 (0.02–0.03)	0.009
Appendix diameter (mm)	7 (7–8)	10 (10–11)	<0.001

CRP: C-reactive protein, WBC: White blood cell count, CI: Confidence interval.

**Table 7:** Modeling results for negative versus complicated AAP.

	AdaBoost	XGBoost	SGB	Bagged CART	Random Forest
Accuracy	84.7	97.3	89.2	91.5	89.8
Balanced Accuracy	83.6	89.1	86.4	91.5	88.5
Sensitivity	88.9	100.0	95.8	91.7	94.4
Specificity	78.3	78.3	76.9	91.3	82.6
PPV	86.5	87.8	88.5	94.3	89.5
NPV	81.8	100.0	90.9	87.5	90.5
F1 Score	87.7	93.5	92.0	93.0	91.9

SGB: Stochastic Gradient Boosting, PPV: positive predictive value; NPV: negative predictive value.

**Table 8:** Variables’ importance for negative versus complicated AAP.

Variables	Variable Importance
Appendix diameter (mm)	100
CRP	71.5
WBC	56.0
Neutrophils	44.4
Monocytes	35.9
Platelets	7.3
Eosinophils	7.3
Lymphocytes	6.9
Basophils	4.3
Immature granulocytes	3.5

CRP: C-reactive protein, WBC: White blood cell count.

**Table 9:** Uncomplicated versus complicated AAP.

Variables (Median (95% CI))	Uncomplicated	Complicated	*p*
Age	11 (11–12)	10 (10–11)	0.003
CRP	7.9 (6.3–9.7)	42.6 (35.2–58)	<0.001
Platelets	274 (264–283)	278 (265–295)	0.277
WBC	13.5 (13.2–14.3)	15.3 (14.7–16.5)	<0.001
Neutrophils	10.7 (10.3–11.5)	12.8 (12.0–13.7)	<0.001
Immature granulocytes	0.05 (0.05–0.06)	0.05 (0.05–0.06)	0.002
Lymphocytes	1.66 (1.55–1.77)	1.25 (1.14–1.4)	<0.001
Monocytes	0.9 (0.84–0.97)	1.1 (1.02–1.18)	<0.001
Eosinophils	0.05 (0.04–0.07)	0.01 (0.01–0.02)	<0.001
Basophils	0.03 (0.03–0.04)	0.02 (0.02–0.03)	0.005
Appendix diameter (mm)	9 (9–10)	10 (10–11)	<0.001

CRP: C-reactive protein, WBC: White blood cell count, CI: Confidence interval.

**Table 10:** Modeling results for uncomplicated versus complicated AAP.

	AdaBoost	XGBoost	SGB	Bagged CART	Random Forest
Accuracy	70.8 (67.0–74.3)	80.0 (76.6–83.0)	75.4 (71.8–78.7)	75.4 (71.8–78.7)	75.4 (71.8–78.7)
Balanced Accuracy	66.5 (63.9–69.0)	78.1 (75.8–80.4)	74.4 (71.9–76.9)	71.0 (68.6–73.4)	71.8 (69.4–74.3)
Sensitivity	50.0 (46.0–54.0)	70.8 (67.0–74.3)	70.8 (67.0–74.3)	54.2 (50.2–58.2)	58.3 (54.3–62.2)
Specificity	82.9 (79.7–85.7)	85.4 (82.3–88.0)	78.0 (74.5–81.2)	87.8 (84.9–90.2)	85.4 (82.3–88.0)
PPV	63.2 (59.2–67.0)	73.9 (70.2–77.3)	65.4 (61.5–69.1)	72.2 (68.5–75.7)	70.0 (66.2–73.6)
NPV	73.9 (70.2–77.3)	83.3 (80.1–86.1)	82.1 (78.8–85.0)	76.6 (73.0–79.8)	77.8 (74.3–81.0)
F1 Score	55.8 (51.8–59.8)	72.3 (68.6–75.8)	68.0 (64.1–71.6)	61.9 (57.9–65.7)	63.6 (59.6–67.4)

SGB: Stochastic Gradient Boosting, PPV: positive predictive value; NPV: negative predictive value.

**Table 11:** Variables’ importance for uncomplicated versus complicated AAP.

Variables	Variable Importance
CRP	100
Eosinophils	42.8
Monocytes	19.1
Age	14.0
WBC	13.8

CRP: C-reactive protein, WBC: White blood cell count.

## Data Availability

The datasets analyzed during the current study are available from the corresponding author upon reasonable request.
